# Framework as a Service, FaaS: Personalized Prebiotic Development for Infants with the Elements of Time and Parametric Modelling of In Vitro Fermentation

**DOI:** 10.3390/microorganisms8050623

**Published:** 2020-04-25

**Authors:** Ka-Lung Lam, Wai-Yin Cheng, Fan Yang, Shaoling Lin, Lijun You, Jiachi Chiou, Hoi-Shan Kwan, Peter Chi-Keung Cheung

**Affiliations:** 1 Food and Nutritional Sciences, School of Life Sciences, The Chinese University of Hong Kong, Shatin, Hong Kong SAR 000000, China; cufns_kocas@link.cuhk.edu.hk (K.-L.L.); nanocheng@link.cuhk.edu.hk (W.-Y.C.); 1155134641@link.cuhk.edu.hk (F.Y.); hoishankwan@cuhk.edu.hk (H.-S.K.); 2 College of Food Science, Fujian Agriculture and Forestry University, Fuzhou 350002, China; shaoling.lin@fafu.edu.cn; 3 School of Food Science and Engineering, South China University of Technology, Guangzhou 510640, Guangdong, China; feyoulijun@scut.edu.cn; 4 Department of Applied Biology and Chemical Technology, Hong Kong Polytechnic University, Hung Hom, Hong Kong SAR, China; jiachi.amber.chiou@polyu.edu.hk

**Keywords:** parametric modeling, biological parameters, composite indicator, selected prebiotic library, structural characterization, 16S amplicon sequencing, parallel screening, structure–property relationship, personalized nutrition

## Abstract

We proposed a framework with parametric modeling to obtain biological relevant parameters from the total probiotic growth pattern and metabolite production curves. The lag phase, maximum increase rate, and maximum capacity were obtained via a 205-h exploratory In vitro fermentation of a library of 13 structural-characterized prebiotic candidates against an exclusively breastfed infant fecal inoculum. We also conducted 16S rRNA amplicon sequencing of the infant fecal inoculum. Moreover, we introduce a robust composite metabolite-based indicator that reflects the eubiosis/dysbiosis of microbiota to complement the conventional microbial markers. In terms of short-chain fatty acid, we discovered that polymeric beta-glucans from barley demonstrated potential as prebiotic candidates, while alpha-glucans as glycogen showed the least dissolved ammonia production. In terms of total probiotic, beta-glucans from oat and mushroom sclerotia of *Pleurotus tuber-regium* showed comparable sustainability when compared to alpha-glucans after 48 h. Being classical prebiotic, galacto-oligosaccharides gave the second-highest metabolite-based indicator, followed by lactose. While limited improvement could be made to lactose and oligosaccharides, polymeric beta-glucans from barley avails more capacity for novel prebiotic development, such as structural modification. We anticipate that more similar parallel screening with the element of time and parametric modeling will provide more novel insights.

## 1. Introduction

Parallel In vitro experiments have been employed to delineate the association between lead compounds (targeted compounds of interest for screening), microbial phenotypes (mostly growth characteristics), and metabolite production in the past decades, with mostly pure bacterium as the sole biologics. The investigation of microbiota has been the topic of the city in the last two decades; together with the advancement of high-throughput technology, hundreds of microbial taxa and metabolites in microbiota could be simultaneously identified [1]. Detection of metabolites currently relies mostly on off-line detection systems from chemical/biological assays to instrumental analysis such as mass spectrometry (MS) and nuclear magnetic resonance (NMR). Confining to In vitro investigation, data of microbial abundance from metagenomics and metabolites from metabolomics have been accumulated but are not easily interpreted, with mostly the raw data and/or the regulation profiles available in databases [2].

Sine time domain investigation provides more insights. In accordance with the surge of microbial community research, commercial analysis solutions, although very limited, have been recently developed, such as the Biolog Microbial Community Analysis with EcoPlates (obtaining the growth characteristics against 31 pre-defined carbon sources). These tools have significantly enhanced the research in compound-microbiota-phenotype “temporal” investigation but are confined mostly to the community growth characteristics (total microbial population increase) instead of their metabolites. While the use of high-ended instruments such as MS and/or NMR provides multiple target detection and high sensitivity, there are considerations in cost, time, and expertise required. Hence, such analyses might not be appropriate for an initial screening. Moreover, targeted analysis assays could sometimes provide higher accuracy, such as enzymatic assays and/or ELISA, due to substrate-specificity of enzymes and/or antibodies.

In view of the classical In vitro exploratory screening (most screening does not employ a multi-omics approach) that has been widely adopted in both research and industry, in order to close the gap of the tri-parties (lead compounds, microbial community phenotypes, and metabolite production “against time”), we proposed a framework that includes two extra analysis procedures: (1) parametric modeling of microbial community growth and targeted metabolite production temporal patterns, and (2) calculation of a “functional” indicator based on metabolites. We also urge scientists to share these extra analysis results for further insights acquisition and meta-analysis. We exemplified this framework with a screening of 13 carbohydrates as potential prebiotics [3]; this framework can be extended to users’ targets of interest.

Parametric modeling excels with the use of several parameters to describe (and, therefore, the capacity to re-construct) the whole model, and notably, these parameters themselves embody biological meanings, such as the lag phase, maximum growth rate, and maximum population increase in logistic and Gompertz functions [4]. Parametric modeling of the bacterial community population pattern, together with some biologically important metabolite production patterns, is essential in filling the gap of knowledge and potentiating further research.

On the other hand, a robust functional indicator based on the production of metabolites by a microbial community is proposed in view of a functional redundancy demonstrated by microbe-based markers [5], while the use of metabolite-based indicators directly captures the functional potential. Complementation of both functional indicators and microbial markers allows a more rigorous conclusion. Moreover, this proposed functional indicator was found to possess extra applicability by showing a relatively similar profile at both 24- and 48-h, and therefore allowing a rough comparison of prebiotic potential at these two time-points, especially when data at either one of the time points were provided.

The choice of short-chain fatty acid (SCFA) profiles as targeted metabolites arose from its representation of microbial saccharolytic potential, and the dissolved ammonia is the “un-assimilated” nitrogen after proteolysis. Under normal situations, any accessible nitrogen will be assimilated as microbial growth, development, enzyme production, and so on. However, nitrogen that is not assimilated will result in microbial bio-conversion, and, frequently, the end-products might not be beneficial to the host. Evidence has suggested that saccharolytic fermentation is more favorable than the proteolytic counter-part [6]. Therefore, we chose ammonia as un-assimilated nitrogen and proposed the SCFAs/NH_3_ ratio as a more relevant microbiota functional indicator.

**Figure 1:**
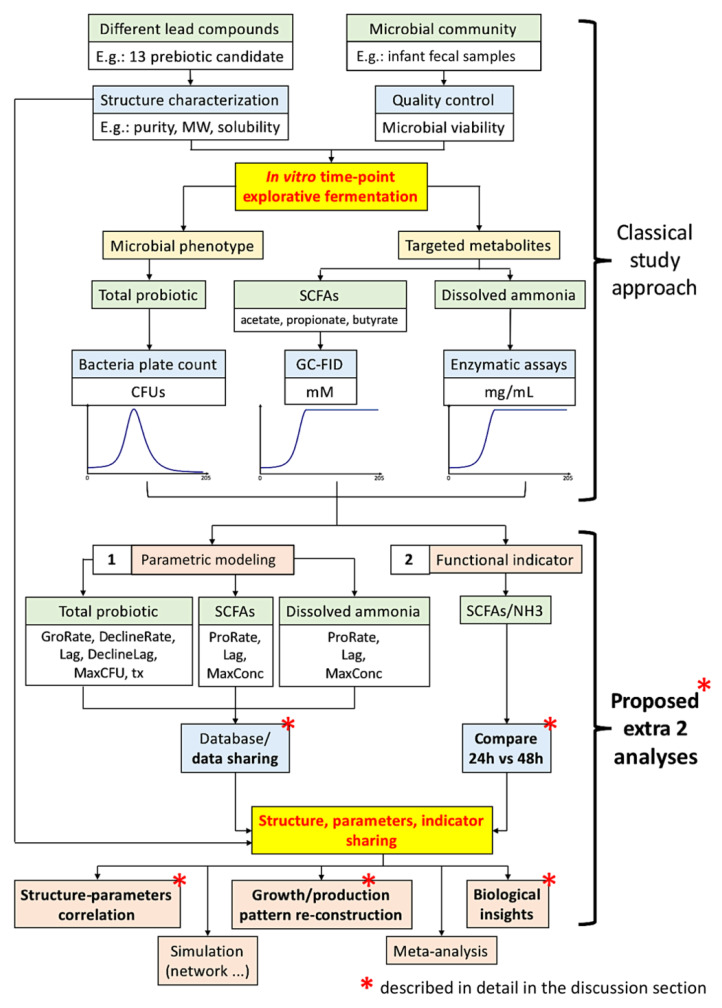
Proposed framework with an extra two analyses procedures together with the classical investigation approach to obtain more in-depth biological insights. The two additional analysis procedures include (1) parametric modeling of the microbial community population pattern and metabolite (short-chain fatty acids, SCFAs, and dissolved ammonia, NH_3_) production patterns; (2) the calculation of a microbiota functional indicator based on metabolites. Database construction and/or data sharing is also important to further potentiate the novel insights obtained and aid meta-analysis. The whole framework can also be adopted for a personalized evaluation of prebiotics, such as for infants. Commensal bifidobacteria are enumerated as total probiotics.

Our framework is summarized in Figure 1. In this study, we pursued an In vitro fermentation of human infant fecal inoculum with a library of 13 selected prebiotic candidates to examine their prebiotic potentials. Total microbial population was quantified using classical bacteria plate count. We further quantify the SCFAs, including total, acetic, propionic, and butyric acids, and dissolved ammonia (NH_3_) profiles of the 13 carbohydrates, along an exploratory 205-h anaerobic fermentation study.

The advancement of omics technology deems personalized nutrition probable, and individuality has progressively highlighted [7]. Individual adults can consume different prebiotic supplements and/or prebiotic products to identify individually optimal products by trial-and-error; however, this trial-and-error approach might not be a conservative approach for new-born infants. While it might not be ethical and safe to directly evaluate the effects of selected prebiotic candidates to infants by feeding (especially in the first several months after birth), the use of parallel In vitro fermentation, with their fecal sample against a library of different prebiotic candidates simultaneously, does no harm and allows a personalized development of prebiotic products and/or prebiotics to be added in infant formula [8], possibly aiming for certain optimal biological function, such as a faster rate of SCFA production or an increase of probiotic bacteria. Our proposed In vitro parallel investigation framework could also serve the purpose of this individual-specific personalized evaluation.

Overall, the objectives of this study are (1) to obtain the basic chemical of structural features of a library of 13 selected carbohydrates as prebiotic candidates; (2) parametrically model the microbial community population, SCFAs (total, acetate, propionate, and butyrate), and ammonia production temporal patterns; (3) the development of a composite metabolite-based (SCFAs/NH_3_ ratio) indicator as a robust functional indicator for microbiota eubiosis/dysbiosis; (4) all data of this research work are provided as Supplementary Materials for users to adopt and adapt, and we urge scientists likewise to share their models and the parameters; (5) identify potential prebiotic candidates that are worth further development based on this In vitro parallel fermentation screening framework; (6) our proposed framework also served the development of personalized prebiotic nutrition, especially for infants. Only with these accumulated data, more complex microbial networks can be modeled/simulated, allowing more in-depth insights.

## 2. Materials and Methods

### 2.1. Materials and Carbohydrates Used

All chemicals were obtained from Sigma-Aldrich (St. Louise, Missouri, USA) unless otherwise specified. Thirteen carbohydrates, including (1) glucose, (2) lactose, (3) sucrose, (4) xylitol (Wako Pure Chemical Industries, Ltd., Osaka, Japan), (5) fructo-oligosaccharides (FOS; Wako Pure Chemical Industries, Ltd., Osaka, Japan), (6) galacto-oligosaccharides (GOS; Oligomate, Yakult Pharmaceutical Industry Co., Ltd., Tokyo, Japan), (7) xylo-oligosaccharides (XOS; Wako Pure Chemical Industries, Ltd., Osaka, Japan)), (8) oat beta-glucan (Oat_bG; Xi’an Rongsheng Biotechnology Co., Ltd., Xi’an, China), (9) beta-glucan extracted from sclerotia of *Pleurotus tuber-regium* (Ptr_bG), (10) barley low viscosity beta-glucans (Barley_bG; Megazyme, Bray, Ireland), (11) glycogen, (12) starch, and (13) inulin, were used as the carbon sources for the microbial fermentation.

Ptr_bG was extracted based on a previous publication [9]. Briefly, sclerotia of macro-fungus *Pleurotus tuber-regium* were oven-dried and pulverized, with the removal of water-soluble fractions three times, followed by 1.0 M NaOH extraction twice; supernatants were pooled together and neutralized using 1.0 M HCl to precipitate the beta-glucan polysaccharide, with repeated washing using Milli-Q water and 80% ethanol and finally freeze-dried after dialysis desalting.

### 2.2. Purity, Solubility, and Molecular Weight Assessment of 13 Carbohydrates

Carbohydrate purity was accessed by elemental analysis; samples were precisely weighed (3–5 mg) in tin boats, tightly folded, followed by instrumental analysis (Elementar rapid OXY cube, Langenselbold, Germany). The molecular weight (MW) distributions were determined by size exclusion chromatography (SEC) (Waters e2695 HPLC equipment system, Milford, Massachusetts, USA) coupled with a refractive index detector (Waters 2414, Waters Inc., Milford, Massachusetts, USA) (40 °C). In brief, TSK gel G6000 PW (30 cm × 7.5 mm i.d., Supelco Analytical, Bellefonte, Pennsylvania, USA) was installed. A range of dextran standards (5, 25, 80, 150, 410, 670 kDa) was used for MW calibration. Samples were dissolved in Milli-Q water (10 mg/mL) and filtered through 0.45-mm filters before injection. Water was applied as a mobile phase at a flow-rate of 0.5 mL/min at 60 °C. Solubility was visually inspected in culture medium after sterilization and denoted as “2” for soluble, “1” for colloidal, and “0” for insoluble.

### 2.3. Infant Fecal Sample Collection and In vitro Fecal Fermentation

Infant fecal samples were collected from a 3-month old baby (3–15 months is known as the microbiota developmental phase [10] and, therefore, essential in choosing individually optimal prebiotics) delivered by natural birth and exclusively breastfed (and, therefore, containing mostly probiotics such as *Bifidobacterium* sp. [11]). Informed consent was obtained from the mother for inclusion before participation in the study. The study was conducted in accordance with the Declaration of Helsinki, and the protocol was approved by the Clinical Research Ethics Committee of the Joint Chinese University of Hong Kong (CREC Ref. No: 2015.206, 14 March 2015). The fecal slurry was stocked in 30% glycerol at −80 °C until use. Both the infant and mother had not taken antibiotics or medicines for the last three months prior to the collection. Viability was checked before fermentation using total bacteria plate count.

The fecal samples were then mixed with the modified medium for colonic broth (mMCB) in a ratio of 1:9 [12], and the 13 carbohydrates were added at a concentration of 2% *w*/*v*. Modified medium for colonic broth contained (per liter) 6.5 g of bacteriological peptone, 5.0 g of soy peptone, 2.5 g of tryptone, 3.0 g of yeast extract, 2.0 g of KCl, 0.2 g of NaHCO_3_, 4.5 g of NaCl, 0.5 g of MgSO_4_·7H_2_O, 0.45 g of CaCl_2_·2H_2_O, 0.2 g of MnSO_4_·H_2_O, 0.005 g of FeSO_4_·7H_2_O, 0.005 g of ZnSO_4_·7H_2_O, 0.4 g of cysteine–HCl, 0.005 g of hemin, 0.005 g of menadione, 0.5 mL of H_3_PO_4_, and 2 mL of Tween 80. There are no carbon sources in mMCB. The fermentation, with a biological triplicate, was performed anaerobically at 37 °C. A negative control group was also explicitly included without any carbohydrate added.

Aliquots (1000 µL) of samples were collected at selected intervals to monitor the total probiotic population, the SCFA profiles, and the dissolved NH_3_ concentration during a period of 205 h. Sampling was made a total of 19 time-points, including 0, 4, 10, 22, 24, 30, 36, 48, 55, 61, 73, 79, 85, 97, 109, 130, 153, 178, and 205 h. Briefly, the collection interval was four time-points on the 1st day; three time-points on the 2nd, 3rd, and 4th days; two time-points on the 5th day; and one time-point on the 6th, 7th, 8th, and 9th days.

Since the investigation was an exploratory study, we did not set an end-point in advance. Instead, we ceased the investigation when all prebiotic candidates showed almost no viable bacteria in total plate count, giving 205 h as an end, i.e., there was a prebiotic candidate that could give an excellent microbial community sustainability to approximate 205 h.

### 2.4. DNA Extraction, PCR, and 16S rRNA Amplicon Sequencing of the Infant Fecal Sample

There was only one infant fecal sample for the 16S amplicon sequencing to evaluate the bacteria composition and abundance [12]. Briefly, DNA extraction was conducted using EZNA Stool DNA Kits (Omega Bio-tek Inc., Norcross, GA, USA) in accordance with the manufacturer’s manual with an enzymatic pre-treatment step (mutanolysin and lysozyme) and a bead-beading step to enhance DNA release from Gram-positive bacteria. 16S V3 region [13] amplicons were PCR generated (New England BioLabs, Ipswich, MA, USA) with a special primer-pair (Probio_Uni: CCTACGGGRSGCAGCAG; and Probio_Rev: ATTACCGCGGCTGCT), gel purified (Qiagen, Venlo, Netherlands), followed by AMPure treatment (Beckman Coulter, Brea, CA, USA). The quality of the DNA amplicons was accessed by Qubit fluorometer (Thermo Scientific, Waltham, MA, USA) and bio-analyzer (Agilent, Santa Clara, CA, USA). The sequencing library was then prepared, followed by emulsion PCR and enrichment. Then DNA sequencing was done using the Ion PGM Sequencing 400 Kit on Ion torrent PGM using Chip 318v2 in the core facilities of the Chinese University of Hong Kong.

### 2.5. Bioinformatic Analysis of 16S rRNA Amplicon Sequencing

After sequencing, the individual sequence reads were filtered by the PGM software to remove low quality and polyclonal sequences. Sequences matching the PGM 3′ adaptor were also automatically trimmed. All PGM quality-approved, trimmed, and filtered data were exported as .sff files. Bioinformatics analysis was done using the Qiime2 version 2020.2 pipeline [14]. Briefly, single-end sequencing raw read file as .fastq was imported (using the manifest approach), denoised, and chimera-removed using DADA2. The SILVA version 132 database was used as a reference for bacterial 16S sequence identity. An in-house classifier was trained with both the SILVA-132 database at a 99% similarity level and the special primer-pair that was used to generate the 16S rRNA amplicons. Taxonomy data were exported at the genera level. The amplicon sequencing data have been submitted to NCBI SRA and are available under BioProject ID: PRJNA611080.

### 2.6. Total Bacterial Plate Count of Probiotics

Total microbial enumeration was performed using plate count [12] as it gives only viable bacteria. Standard total plate count (TPC) was done using TOS propionate agar (TPA) with mupirocin (Sigma-Aldrich (St. Louise, Missouri, USA)), under anaerobic conditions in triplicate, using the standard aseptic culture procedure of serial dilution with sterile PBS. The total probiotic population in the fermentation mixture was expressed as colony-forming units (CFUs). Breast-fed infants contain in gut mostly probiotic species. In addition, TPA (TOS propionate agar) were specifically designed and prepared for a selective enumeration of presumptive *Bifidobacteria* sp. It contains highly purified “galacto-oligosaccharides”, which are one of the most excellent bifidobacterial growth substances. Therefore, we enumerated TPC as an indicator of total probiotics [11].

### 2.7. Short Chain Fatty Acid Quantification

SCFAs were extracted and analyzed using gas chromatography–flame ionization detection (GC–FID) [15]. In brief, 600 μL fermentation mixtures were acidified with 25% metaphosphoric acid. Then, 350 μL of supernatant (after centrifugation) was extracted with 600 μL of diethyl ether twice and 0.22 μm filtered. Internal standard: 30 μL of 4-methyl-valeric acid (100 mg/mL) was added. External standards include acetic, propionic, butyric, valeric, and caproic acids (SCFA standards kit, Alltech Inc., Nicholasville, Kentucky, USA). Instrumental analysis was conducted with HP 6890 GC system (Agilent Technologies, Santa Clara, California, USA) equipped with a Quadrex 007-FFAP capillary column (30 m × 0.25 mm i.d.; 0.25-μm film, Quadrex Corporation, Woodbridge, Connecticut, USA) with a 0.5 mL/min hydrogen flow rate. The oven was programmed at an initial temperature of 80 °C with a hold of 5 min, followed by a temperature rise of 5 °C/min to 180 °C with a final hold of 5 min at 220 °C. Two microliters of samples were injected, with signal detection by flame ionization. The amount of the SCFAs was expressed in mM.

### 2.8. Dissolved Ammonia Determination

Ammonia was determined using an ammonia rapid assay (Megazyme, Bray, Ireland, Cat. no.: K-AMIAR). It is a highly specific assay involving the enzymatic conversion of 2-oxoglutarate, NADPH, and dissolved ammonia into L-glutamic acid, NADP+, and water under the action of glutamate dehydrogenase. Originally, the assay was not developed for NH_3_ determination of In vitro fermentation, and, therefore, protocol optimization was executed.

Final optimized experimental protocol: 100 µL fermentation samples were centrifuged at 14,000 rpm (Spectrafuge 24D, Labnet, Woodbridge, New Jersey, USA) for 5 min, then 2 µL of supernatants (larger volume might result in assay saturation), ammonia standard (0.04 mg/mL) and water as blank were added with 30 µL buffer and 20 µL NADPH with 208 µL PBS (pH 9.8). Absorbance (A1) was taken at 340 nm with a microplate reader (Molecular Devices SpectraMax Plus, San Jose, California, USA). Then, 2 µL glutamate dehydrogenase was added to each well and allowed to react for 5 min, followed by second absorbance (A2) at 340 nm. The final ammonia concentration (mg/mL) was calculated as

Concentration of NH_3_ = (Sample [A1 − A2] / Standard [A1 − A2]) × mg/mL Standard, (1)

### 2.9. SCFAs/NH_3_ Ratio Determination

Total SCFAs (by summation of acetic, propionic, and butyric acids) and dissolved NH_3_ concentration were quantified. Since the total SCFAs and NH_3_ had different units (mM and mg/mL, respectively), min–max normalization was applied to confine a range of (1, 10).

Under most circumstances, the normalization step transforms data into a range of (0, 1); however, since we have the next step of SCFAs/NH_3_ division, division with 0 will gave an undefined value, while others have suggested the addition of a very small number (such as 0.00001) to each value before scaling, this will occasionally give an extremely large or extremely low value when the small value is divided or being divided, and therefore, again, not biologically relevant.

We mimicked the (0, 1) range as a (1, 10) range:

Scaled total SCFAs or NH_3_ = a + ((X − X_min_)(b − a))/(X_max_ − X_min_), (2)

where X is the SCFAs or NH_3_ concentration; a is the lowest ceiling of range, noted as 1; b is the highest ceiling of range, noted as 10; X_min_ is the minimum total SCFAs or NH_3_ concentration; X_max_ is the maximum total SCFAs or NH_3_ concentration.

Then, the SCFAs/NH_3_ ratio was calculated as a roust metabolite-based indicator for eubiosis/dysbiosis for each time-point:

Robust functional indicator = (Scaled total SCFAs)/(Scaled NH_3_), (3)

### 2.10. Parametric Modelling of SCFA Production and NH_3_ Temporal Production Patterns

The R package “grofit” [4], version 1.1.1, was adopted to fit the parametric models of SCFA production and NH_3_ production curves. We confined the models to either logistic or Gompertz as these two models require minimally three parameters. Optimal models were chosen based on the Akaike information criterion (AIC) scores.

The logistic model:

y(t) = A/(1 + exp(4μ/A(λ − t) + 2)),(4)

The Gompertz model:

y(t) = A × exp[−exp(((μ × e)/A(λ − t)) + 1),(5)

where y(t) is the concentration of SCFAs or NH_3_ respectively (same as the following) at time t; A is the maximum production capacity, the MaxConc (mM or mg/mL); μ is the maximum production rate, the ProRate (mM/h or mg/mL/h); λ is the production lag phase, Lag (h); e is the base of the natural logarithm; t is the time (h).

The modeled parameters of SCFA (including total, acetate, propionate, and butyrate) production and NH_3_ are listed in Tables S1 and S2, respectively, with the fitted models and values of Lag, ProRate, and MaxConc expressed as means with standard deviations.

### 2.11. Parametric Modelling of Total Probiotic Population Pattern

Direct modeling fails in capturing the whole microbial population pattern due to the presence of the decline phase. Instead of adopting more complex models [16], we maintained the logistic and Gompertz functions [4] to model the microbial community population pattern to obtain the minimal number of parameters, requiring several extra steps:

1. identify the immediate population maximum in CFU and noted the time as tx (tipping point);
2. keep all the CFU after time tx identical to the CFU at time tx;
3. perform “grofit” [4] analysis to obtain model and parameters;
4. keep all the CFU before time tx identical to the CFU at time tx;
5. the variable time t of the parametric models fitting using “grofit” in is now noted as (205 − t).

The total probiotic population pattern is now defined with two situations:

Situation 1 for t < tx, the logistic model is

y(t) = A/(1 + exp(4μ/A(λ − t) + 2)),(6)

and the Gompertz model is

y(t) = A × exp[−exp(((μ × e)/A(λ − t)) + 1),(7)

where y(t) is the total probiotic population (CFU); A is the maximum population capacity, the MaxCFU (CFUs); μ is the maximum population increase rate, the GroRate (CFU/h); λ is the lag phase, Lag (h); e is the base of the natural logarithm; t is the time (h).

Situation 2 for t > tx, the logistic model is

y(t) = A/(1 + exp(4μ/A(λ − (205 − t)) + 2)),(8)

and the Gompertz model is

y(t) = A × exp[−exp(((μ × e)/A(λ − (205 − t))) + 1),(9)

where y(t) is the total probiotic population in CFU; A is the maximum capacity, the DeclineMaxCFU (in CFUs); μ is the maximum population decline rate, the DeclineRate (CFU/h); λ is the lag phase, DeclineLag (h), where (205-DeclineLag) represents the time that the total probiotic population is increased and then declined to level off; e is the base of the natural logarithm; t is the tipping point, time after tx (h).

**Figure 2:**
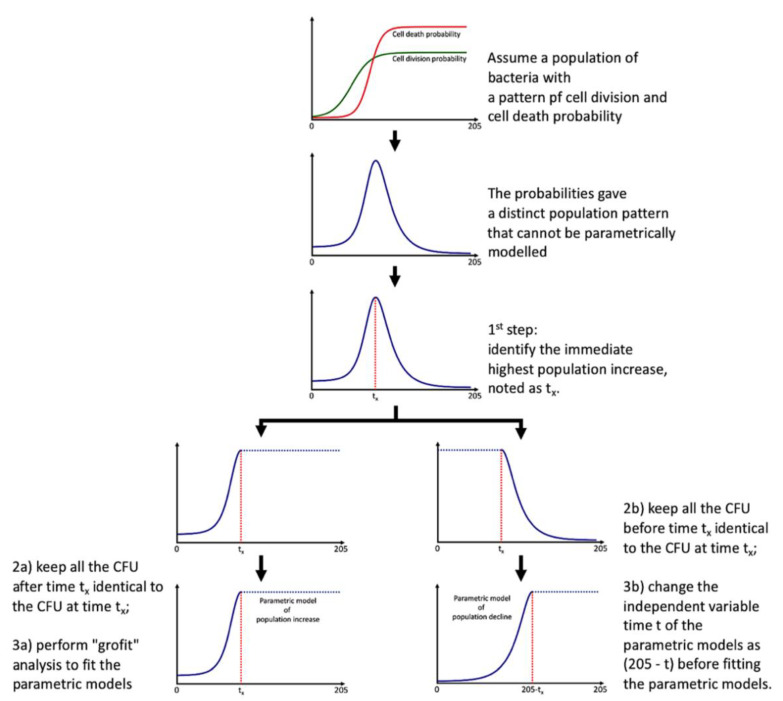
Parametric modeling of total probiotic population pattern with a decline phase. The use of logistic and/or Gompertz functions cannot capture the microbial community growth pattern due to the presence of a decline phase. Instead of employing other complex models that include the decline phase, we insisted on the use of either the logistic and/or Gompertz functions with extra steps, composed of the identification of tipping point tx, followed by the modeling of the logistic and Gompertz functions separately on the exponential increase part and the decline phase part after mirroring. Still, all these parameters obtained possess biological meanings. Commensal bifidobacteria are enumerated as total probiotics.

The essence of this modeling is shown in Figure 2. We obtained an additive model with two parametric functions (could be logistic and/or Gompertz) with a tipping point of tx. The additive model might not be smooth at tx; scientists could gap the two additive models (e.g., using a logistic function), but this is not always necessary. By using the additive model, we limited the total probiotic population pattern only with seven biologically relevant parameters with an extra procedure to compare time t with tx (i.e., if t > tx, then Situation 2 applies; otherwise, Situation 1 applies). The models and parameters of the total probiotic population pattern are shown in Table S3 with the fitted models and values of GroRate, Lag, MaxCFU, tx, DeclineRate, DeclineLag, DeclineMaxCFU expressed as means with standard deviations.

Particularly, the tipping point tx could be exploited to evaluate the time needed for the total probiotic to reach maximum. On the other hand, the DeclineLag of the decline phase could represent the total sustainability of the prebiotic, by calculating the value of (205-DeclineLag), representing the maximum time for a prebiotic to sustain the growth of total probiotic. A negative DeclineLag represents that the bacterial population had not yet reached zero after 205 h; therefore, both tx and (205-DeclineLag) serve as useful indicators for sustainability evaluation.

### 2.12. Statistics and Exploratory Analysis

Unless otherwise specified, R-3.6.0 [17] was used, and ANOVA with TukeyHSD posthoc adjustment (CI = 0.95) was employed. Various R packages were used, including “ggplot2”, “RColorBrewer”, “PerformanceAnalytics” (Pearson’s correlation), “grofit” (parametric modelling), “reshape2”, “tidyr”, “proxy”, and “factoextra” (PCA biplot).

## 3. Results

### 3.1. Structural Characterization of 13 Carbohydrates

All the 13 carbohydrates were either soluble or in a colloidal state after autoclaving in mMCB, and they all supported microbial growth. The negative control sample without carbohydrates showed no bacteria growth and dropped to 0 CFUs after 10 h anaerobic incubation.

The structural characteristics of the 13 carbohydrates are shown in Table 1. The elemental analysis suggested that Oat_bG had 2.12% N (elemental nitrogen), which represented 12.4% protein (assuming a 5.83 nitrogen conversion factor for oat [18]). Ptr_bG and Starch had 2 peaks in their size exclusion chromatography (SEC) spectra. By considering only the peak with the highest MW, Starch had the highest MW of 1,088,329 kDa, followed by Glycogen, 31,824 kDa. The MW of beta-glucan, including Barley_bG (277 kDa), Ptr_bG (133 kDa), and Oat_bG (1.6 kDa), was also determined by SEC. Mono-saccharide, di-saccharides, and oligo-saccharides showed a MW of less than 2.7 kDa. These structural differences in the carbohydrates could account for their differences in affecting total bacterial community population and differences in microbial metabolite production.

### 3.2. 16S Amplicon Sequencing of Infant Fecal Sample

**Figure 3:**
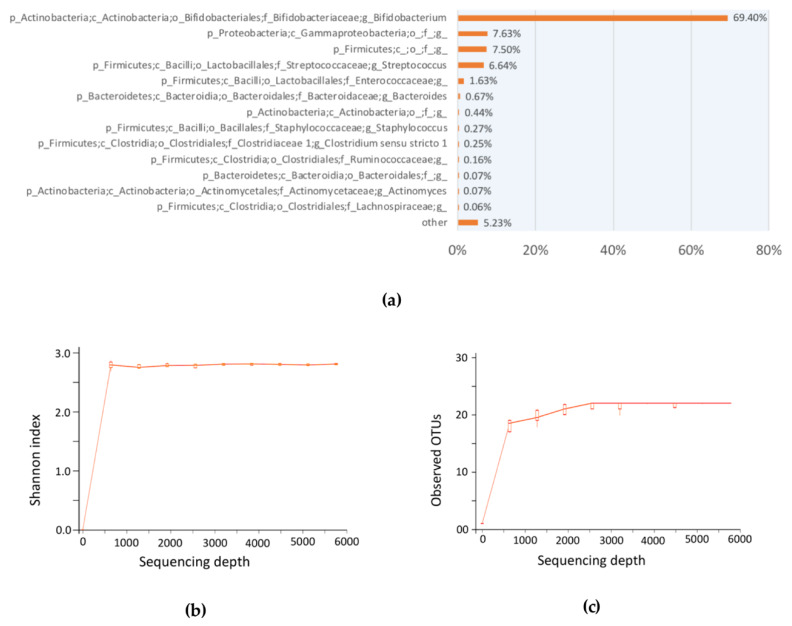
Qiime2 analysis result of 16S amplicon sequencing from infant fecal sample before In vitro fermentation investigation. (**a**) The relative bacteria abundance after 16S amplicon sequencing of infant fecal samples before fermentation. Only the genera level is extracted and shown. Most of the bacteria present in the infant fecal samples were *Bifidobacterium* sp. and, therefore, commensal bifidobacteria are enumerated as total probiotics; (**b**) alpha diversity analysis of Shannon index; (**c**) alpha diversity analysis of observed OTUs (operational taxonomic units).

We have additionally conducted the 16S amplicon sequencing conjointly with TPC to evaluate the bacterial composition and abundance of the infant fecal samples before In vitro fermentation at time 0 against the 13 selected carbohydrates. Figure 3a showed the identification and the relative abundance of the bacteria in the genera level, in descending order, except the group “other” that included reads to be matched to bacteria domains but not further taxonomy levels. After denoise and chimera removal, we obtained a total of 17,364 reads for SILVA-v132 taxonomy identification at 99% similarity.

As shown in Figure 3a, most of the bacteria identified were *Bifidobacterium* sp. (69.40%), accounting for approximately 70% of the bacteria before temporal In vitro fermentation study, followed by a class of *Gammaproteobacteria* (7.63%), the *Firmicutes* phylum (7.50%), and *Streptococcus* sp. (6.64%). Particularly, *Bacteroides* sp. was also found to be 0.67%; *Bacteroides* sp. was identified to be enriched in diet with “complex” polysaccharides [19,20], such as after the introduction of solid food to infants’ diet. Instead of complex carbohydrates, the major carbohydrate constitutes in human breast milk are lactose and human milk oligosaccharides (HMOs) [21], leading to a lower *Bacteroides* sp. abundance, while HMOs was found to selectively enrich *Bifidobacterium* sp [22]. Alpha diversity analysis, both the Shannon index (Figure 3b) and observed OTUs (operational taxonomic units) (Figure 3c), has also confirmed a sufficient sequencing depth, showing this sequencing should capture most of the bacteria taxa in the fecal sample. Also, the diversity of microbial genera identified is low, albeit a total of 17,364 reads sequenced for this sample.

### 3.3. Patterns of Total SCFAs, Dissolved NH_3_ Production, and Total Probiotic Population

Total SCFAs were calculated by the summation of acetate, propionate, and butyrate as the concentration of valeric and caproic acids were negligible (Figure S1). Most SCFAs were eluted with a retention time between 7 to 17 min.

**Figure 4:**
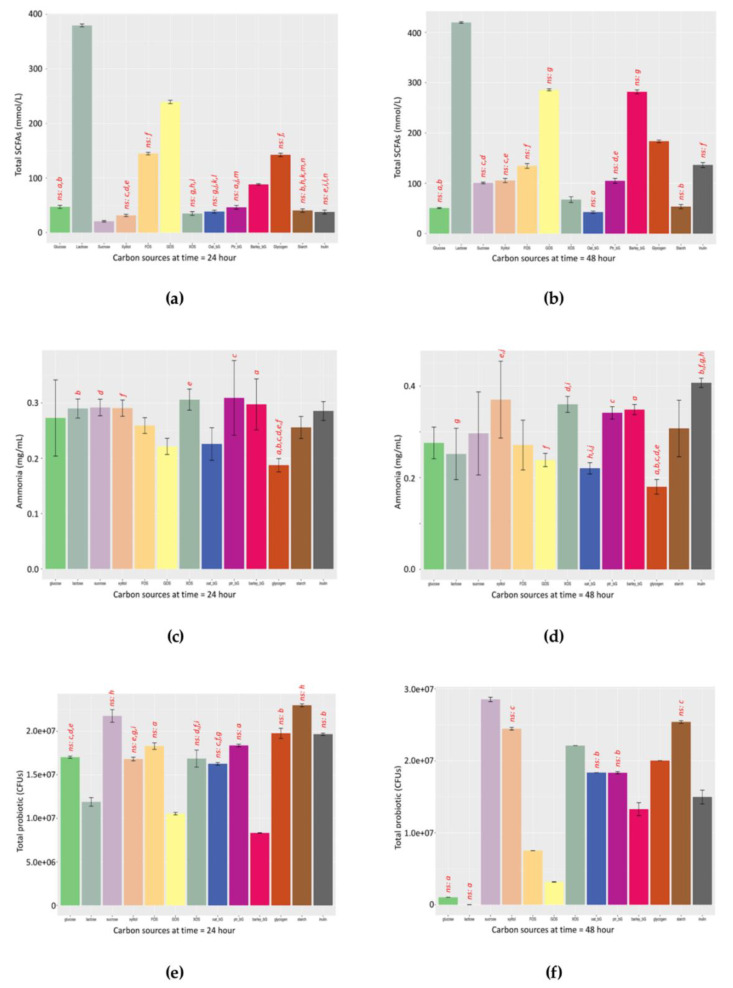

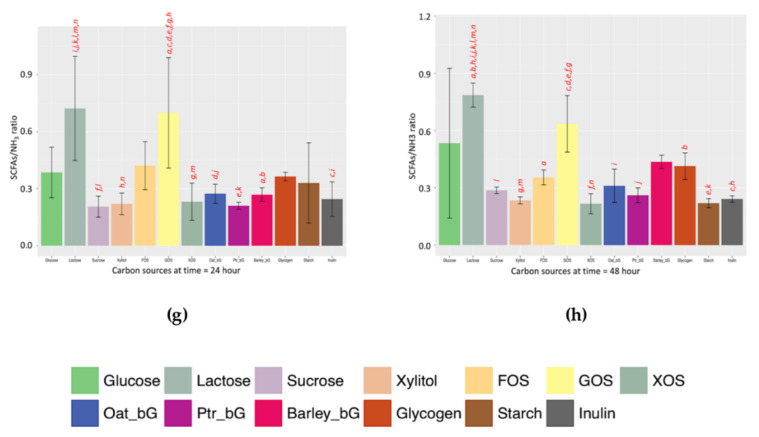
Total short-chain fatty acids (mM), dissolved ammonia (mg/mL), and total probiotic population (CFUs) produced by fermentation against the 13 carbohydrates and the SCFAs/NH_3_ ratio at 24 and 48 h. (**a**) Total short-chain fatty acids (SCFAs) at time = 24 h; (**b**) total short-chain fatty acids at time = 48 h; (**c**) total ammonia concentration (NH_3_) at time = 24 h; (**d**) total ammonia concentration at time = 48 h; (**e**) total probiotic population at time = 24 h; (**f**) total probiotic population at time = 48 h, and commensal bifidobacteria are enumerated as total probiotics; (**g**) SCFAs/NH_3_ ratio at time = 24 h; (**h**) SCFAs/NH_3_ ratio at time = 48 h. ANOVA with Tukey HSD posthoc adjustment (CI = 0.95) was used, paired-letters represented *p*-value < 0.05; “ns” denoted pairs with differences that are “non-significant” while all other pairs are statically significant to avoid over-crowded superscripts.

Since most In vitro fermentation employed 24-h and/or 48-h endpoints, data of these two time-points were extracted for discussion. Figure 4a shows the total SCFAs produced after 24 h with Lactose giving the highest and Sucrose giving the lowest concentrations, while Figure 4b shows the total SCFAs produced after 48 h, with Lactose still the highest and Oat_bG being the lowest. Furthermore, Figure 4b shows that Barley_bG achieved a similar total SCFA with GOS after 48 h, but not 24 h. The individual acetate, propionate, and butyrate profiles at 24 and 48 h are summarized in Figure S2.

NH_3_ represents un-assimilated nitrogen, and less NH_3_ is more desirable. Figure 4c shows NH_3_ production after 24 h, with the lowest NH_3_ production found in Glycogen, followed by GOS, Oat_bG and FOS, while Glycogen remained the least, followed by Oat_bG, FOS and Lactose after 48 h (Figure 4d).

The total probiotic population pattern is represented by an additive model. Usually, we discuss the Lag, GroRate and MaxCFU during the population increase phase. Figure 4e shows that Starch and Sucrose gave the highest MaxCFU, with Barley_bG having the least after 24 h. Figure 4f shows that Sucrose and Starch remained the highest, but Glucose and GOS failed to sustain probiotic growth, with almost no probiotic survival in Lactose. It was postulated that simple carbohydrates were too rapidly metabolized and, therefore, failed in sustaining the probiotic population, albeit providing a relatively faster utilization rate.

All the raw data of total SCFAs, NH_3_ production, and total probiotic increase at 24 and 48 h are summarized in Table S4, expressed as means with standard deviations.

### 3.4. Biological Insights from Exploratory Analysis Based on the Modelled Parameters

Since all the modeled parameters have biological meanings, they can be directly compared to gain novel insights. Moreover, these parameters could be correlated among each other and/or against other data such as structural data and/or the proposed functional metabolite-based indicator.

**Figure 5:**
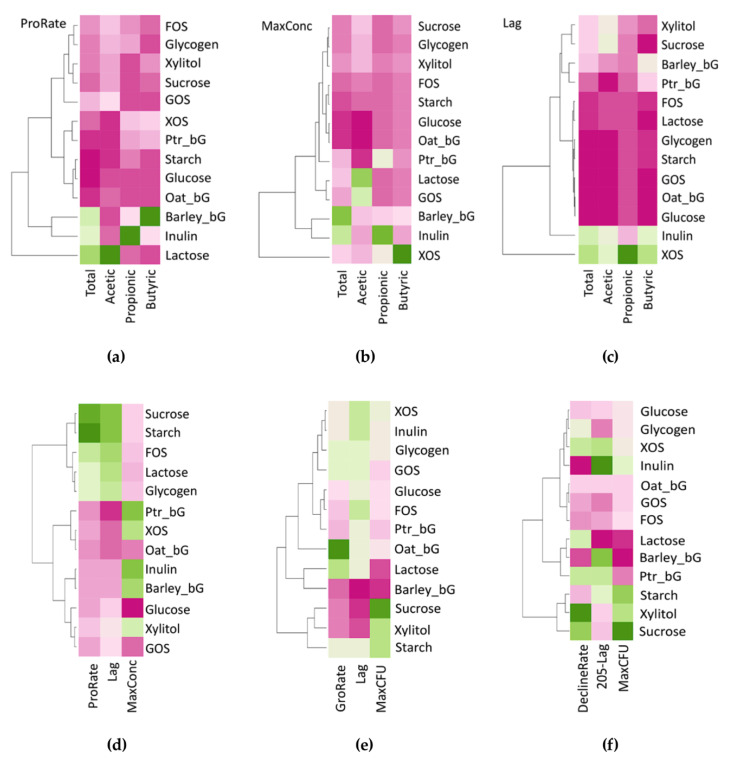
Heatmap showing short-chain fatty acid production characteristics, dissolved ammonia production characteristics, and total probiotic population pattern characteristics during the 205-h fermentation of carbohydrates by infant fecal inoculum. (**a**) Maximum SCFA production rates; (**b**) increase of total SCFA concentration; (**c**) lag time; (**d**) production characteristics of dissolved ammonia; (**e**) population increase characteristics of total probiotic population pattern; (**f**) population decline characteristics of total probiotic population pattern. Green color stands for high while pink color stands for low. Commensal bifidobacteria are enumerated as total probiotics.

A direct comparison of parameters could be explored using a hierarchically clustered heatmap, as in Figure 5. Hierarchical heatmap analysis was performed to visualize the clustering and magnitudes of the model parameters. Lactose, Inulin, and Barley_bG produced higher total SCFAs ProRate with Lactose majoring in acetic, Inulin in propionic, and Barley_bG in butyric acid (Figure 5a). MaxConc of acetic acid arose from GOS and Barley_bG, propionic acid from Inulin, and butyric acid from XOS (Figure 5b), while XOS and Inulin gave higher Lag in SCFA production (Figure 5c). NH_3_ parameters are shown in Figure 5d with Sucrose and Starch giving higher ProRate and Lag. The least NH_3_ MaxConc was observed in Glucose, GOS, and Oat_bG. The total probiotic population patterns were composed of two phases, the increase phase and the decline phase. As for the increase phase (Figure 5e), Oat_bG and Lactose gave higher GroRate, while Sucrose also contributed to the higher MaxCFU with Barley_bG, Sucrose, and Xylitol giving the lower Lag. On the other hand, Inulin and Barley_bG gave a higher value of (205-Lag) in the decline phase (Figure 5f), nurturing longer fermentation sustainability.

**Figure 6:**
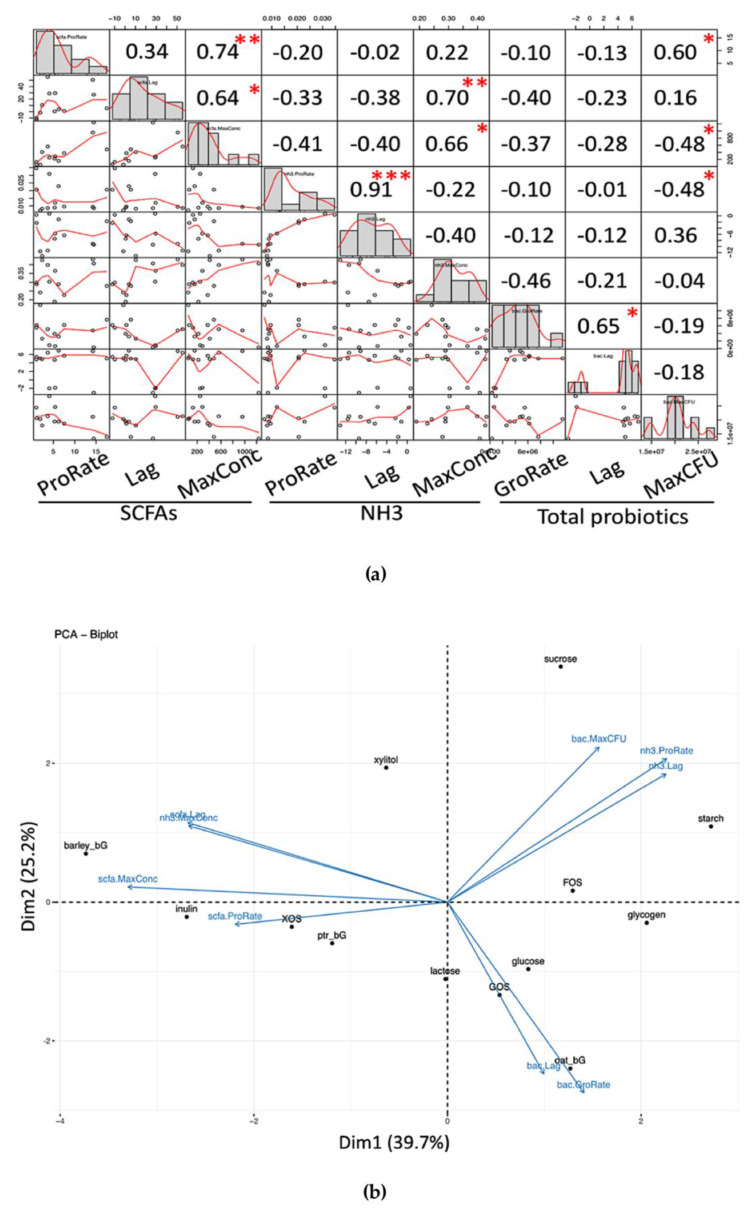
Pearson’s correlation of parametrically modeled parameters and PCA biplot for biological insights. (**a**) Pearson’s correlation of modeled parameters of total probiotic (bac) population pattern, total short-chain fatty acids (SCFAs) production pattern and dissolved ammonia (NH_3_) production pattern. Numbers refer to the Pearson R-squared value while * denotes *p*-value < 0.05; ** denotes *p*-value <0.01; and *** denotes *p*-value < 0.001. (**b**) Exploratory PCA biplot of the modeled parameters and the 13 carbohydrates.

We also exemplified the use of these growth parameters to gain knowledge from Pearson’s correlation, as shown in Figure 6a and the PCA biplot in Figure 6b.

All parameters were correlated (Pearson’s) among each other in Figure 6a. In SCFAs, significant correlations included ProRate increased with MaxConc, and Lag increased with MaxConc. In NH_3_, ProRate increased with Lag. In total probiotic, GroRate increased with Lag. In summary, beneficial phenomena included: (1) NH_3_ ProRate increases with Lag, as prolonging the lag phase would give a lower production rate; (2) higher ProRate of SCFAs allows higher MaxConc of SCFAs; (3) short Lag of SCFAs leads to a lower concentration of MaxConc of NH_3_.

PCA biplot was also performed (see Figure 6b). Three groups were observed: Group 1—Glucose, Lactose, GOS, and Oat_bG contributed more to the probiotic GroRate and probiotic Lag; Group 2—Barley_bG, Inulin, XOS, Ptr_bG contributed more to the SCFAs Lag, SCFAs ProRate, SCFAs MaxConc, and NH_3_ MaxConc; and Group 3—Sucrose, Starch, and FOS contributed more to the probiotic MaxCFU, NH_3_ Lag, and NH_3_ ProRate.

## 4. Discussion

### 4.1. The Use of Viable Bacteria Culture and 16S Amplicon Sequencing

We employed the use of the TPC method to approximately enumerate the total probiotic population. There are two justifications for this approximation: first, most of the bacteria in breastfed-only infant fecal sample was found to be enriched with *Bifidobacterium* sp. [11]; likewise, our 16S amplicon sequencing results suggested the same (Figure 3); second, the use of TOS propionate medium selectively cultivate bifidobacteria in TPC bacteria CFU enumeration. In the discussion of total probiotics that serve as an example of the proposed framework, commensal bifidobacteria were used due to their abundance in the infant microbial community. Of course, other targeted probiotics apart from bifidobacteria could be chosen in other studies of adults.

While the application of 16S amplicon and/or metagenomics sequencing grants more complete profiling (both identity and abundance) of almost all bacteria taxa existed in a community, this method is still not yet universally applied in biotechnology industries and commercial settings, not to mention the inclusion of timepoint investigation, though there is a very recent laboratory effort to study the growth rate (but not the metabolite production parameters) in microbiome using continuous changestat cultivation [23]. The development of a parametric investigation of microbiome temporal patterns is still at its very infancy. Moreover, from a commercial perspective, there are considerations of regulations and standardized methods on the determination of probiotic strains. For example, there are ISO methods such as ISO 7889:2003 (IDF 117:2003) and ISO 29981:2010 (IDF 220:2010) to enumerate presumptive probiotic using CFU technique. The FDA has also allowed CFUs as the measure of dosage strength for new dietary ingredient (NDI) notifications and Generally Regarded as Safe (GRAS) designations. Alternatively, users could choose to use quantitative PCR (qPCR) to enumerate selected probiotics, in-between the TPC and sequencing approaches. qPCR allows the quantification down to strain level, but an enumeration on the genus level might need a universal primer-pair or probe, such as for *Bifidobacterium* sp. and *Lactobacillus* sp.

Users may wish to adopt the 16S amplicons sequencing approach in determining the growth parameters such as Rate, Lag, and MaxCFU (which might be noted as other units apart from CFUs). There are four considerations: first, there is a requirement of sufficient timepoints for the parametric modeling to operate; otherwise, model fitting will fail and no growth parameters will be identified; second, with the necessity of enough timepoints, there is a consideration in the cost-effectiveness; third, absolute abundance instead compositional relative abundance (absolute amount but not percentage) is needed for parametric modeling, such as the use of spike-in DNA [24]; forth, while most of time-domain 16S amplicon sequencing employ statistical techniques in time-series analyses [25,26] such as autoregressive integrated moving average (ARIMA) and Bayesian hidden Markov (HMM) models, the parameters in equations might not necessary embody biological meanings. Our parametric “additive” modeling approach in Figure 2 should be effective in capturing both exponential and decline phases of most taxa in community, but the determination of the tipping point is not automated; program/scripts could be developed in future for automatic parametric modeling of hundreds of taxa by 16S amplicon and/or metagenomics sequencing.

In a nutshell, the choice of probiotic enumeration method for parametric modeling is suggested to be based on the objectives of investigation (such as the level of granularity as in strain or genus levels) and the feasibility in performing the parametric modeling (otherwise, there is no lag phase, maximum growth rate, and maximum population increase). However, most of the enumeration methods could be incorporated into our proposed framework.

### 4.2. The Proposed Two Extra Analysis Procedures

As shown in Figure 1, classical investigation adopts the screening of different lead compounds against the microbial community against time to obtain the microbial community population and targeted metabolite production patterns. Directly published as findings, these temporal patterns can be visually compared, such as the saturation level and the degree of slope inclination; however, visual inspection is not quantitative; hence, explorative analysis such as principal component analysis (PCA) biplots and correlation (such as Pearson’s) cannot be conducted. Moreover, these two additional analysis procedures do not require extra laboratory work, but the benefits are enormous.

The major challenge of our proposed approach is the generation of time-point data that is sufficient for parametric modeling, which could be laborious without automation. Time-point metabolite concentration determination still awaits a complete automation establishment to both instrumental and data analyses, although some semi-automation of metabolite determination has been achieved [27].

### 4.3. Database Construction and/or Data Sharing

One of the objectives of this study is to parametrically model the total probiotic population, different SCFA production, and ammonia production patterns with a minimal number of parameters. This was achieved with the data provided, as shown in Table S1 (total, acetate, propionate, butyrate), Table S2 (dissolved ammonia), and Table S3 (total probiotic). Specifically, we have to emphasize that these data in Tables S1–S3 are different from In vitro fermentation of pure probiotic culture, due to the presence of multiple microbes and their collective interactions inside a community; these data are individually specific.

Furthermore, only these parameters and the logistic and/or Gompertz functions employed are necessary, significantly reducing the hindrance in data sharing (volume of data), i.e., from 13 × 19 × 3 × 6 = 4446 data points (13 carbohydrates, 19 time-points, biological triplicates, total 6 patterns) to 13 × 3 × ((5 × 4) + 8) = 1092 data points (13 carbohydrates, triplicate, SCFAs/NH_3_ has 3 parameters and total probiotic has 7, together with the fitted model), an almost 4-fold reduction in just one investigation, and more importantly, these parameters could be directly compared as they themselves imposed biological meanings.

### 4.4. Re-Construction of Temporal Patterns

With all the parameters needed and the logistic and/or Gompertz functions specified (Tables S1–S3), users can easily reconstruct the microbial community population and metabolite production temporal patterns; any further modeling (such as non-parametric modeling [28] and other novel temporal data analysis [29]) based on the original patterns could then be performed.

### 4.5. SCFAs/NH_3_ as a Robust Functional Indicator of Eubiosis/Dysbiosis

The advantages of the SCFAs/NH_3_ ratio serving as a robust functional indicator lie in two points. The first aspect is the biological rationale that total SCFAs directly represent the saccharolytic potential; the higher is deemed beneficial [6]. On the other hand, a higher concentration of digested but not assimilated nitrogen (as ammonia) after proteolysis is considered a blight; the less, the better. Microbial abundance has been massively investigated using sequencing [1] and/or the micro-array approach [30], and numerous microbial markers are identified after correlation analysis. However, some of these identified microbial markers are controversial as microbes demonstrate functional redundancy in a microbial community [31]; complementation of both metabolite-based functional indicators and microbial markers will allow a more rigorous conclusion.

The second point lies in the robustness of the SCFAs/NH_3_ indicator. By “robustness”, the SCFAs/NH_3_ indicator gives similar profiles at both 24 and 48 h, thus allowing a rough comparison. For example, by comparing the left panel of Figure 4g (24 h) and right panel of Figure 4h (48 h), the pattern of the SCFAs/NH_3_ ratio of these two time-points is more similar than the rest.

### 4.6. SCFAs/NH_3_ Ratio at 24 and 48 h

The patterns of the functional indicator along the 205-h fermentation against the 13 prebiotic candidates are shown in Figure S3a. The total SCFA distribution (Figure S3b), dissolved NH_3_ distribution (Figure S3c), and functional indicator distribution (Figure S3d) of the 13 prebiotic candidates at 24 and 48 h were also included.

We also adopted similar statistics, as shown in a previous publication [32], to evaluate the “robustness” of the SCFAs/NH_3_ indicator (Table 2). Distribution statistics showed that the difference between 24 and 48 h (|slope|) gave the SCFAs/NH_3_ ratio the least dispersion ((|slope|.sd/|slope|.mean). The distribution of the ratio depends on the real data; parametric and non-parametric test could be chosen accordingly. We evaluated both the Wilcoxon rank sum test and *t*-test to the difference in the data distribution at 24 and 48 h. The SCFAs/NH_3_ ratio gave both the highest *p*-value, confirming more area overlap in distribution. Correlation analysis of both Spearman’s and Pearson’s of 24- and 48-h data showed that SCFAs/NH_3_ gave the highest Spearman rho and Pearson R-squared values and the least *p*-values. Finally, we evaluated the similarity of the data at 24 and 48 h with Euclidean, Manhattan, and cosine distance. Euclidean distance considers both magnitude and direction, while the Manhattan distance considers the absolute distance and cosine distance considered only the direction. SCFAs/NH_3_ again gave the highest similarity values in all the three cases.

### 4.7. Structure-Parameters Correlation for Biological Insights

The structural data in Table 1 are correlated with different parameters to obtain structure–phenotype relationships [33]. Selected structural characteristics included molecular weight (MW) peak count, MW of the first eluted peak, solubility, and nitrogen% (N) as impurities. Selected parameters included GroRate and ProRate of both total SCFAs and NH_3_. The functional indicator as SCFAs/NH_3_ ratio at 24 and 48 h was also explored.

**Figure 7:**
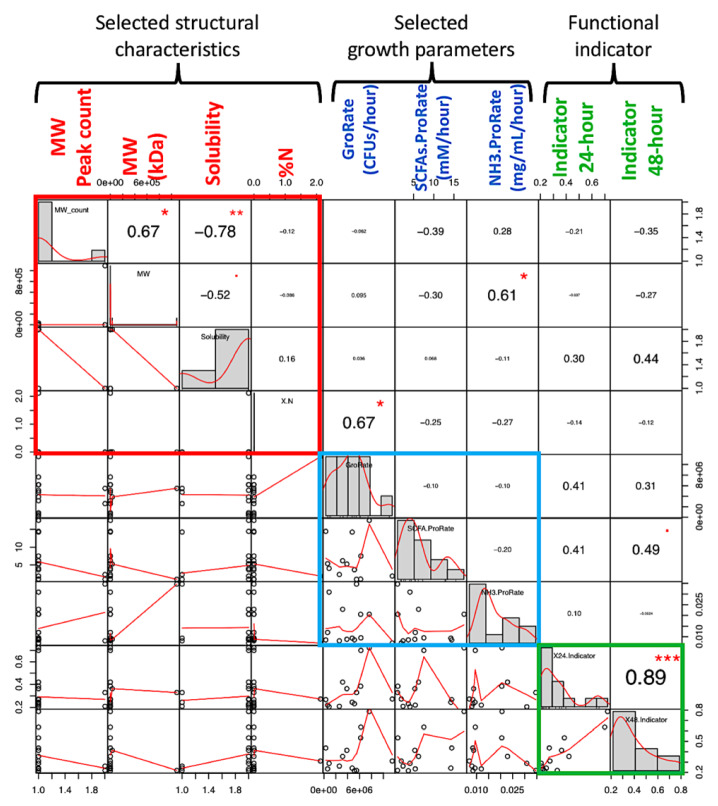
Exemplified selected structure characteristics, selected parameters, and functional indicator correlation analysis. Pearson’s correlation was performed. Selected structural characteristics include molecular weight peak count, molecular weight of first peak, solubility by visual inspection, and % nitrogen (N) as impurities. Selected parameters included GroRate and ProRate of total SCFAs and dissolved ammonia. Functional indicator (SCFAs/NH_3_) at both 24 and 48 h were also included. Significant correlation between structure and parameters were found to be (1) maximum total microbial population increase rate positively correlates with % nitrogen, and (2) dissolved ammonia production rate positively correlates with molecular weight. “*” denotes *p*-value <0.05; “***” denotes *p*-value <0.001.

Pearson’s correlation was performed to assess the relationship between structural data and selected growth parameters and the SCFAs/NH_3_ indicator (Figure 7). A significant correlation between structure and parameters was found to be (1) total probiotic GroRate positively correlates with % nitrogen (N) (such as protein impurities of the carbohydrate samples), and (2) NH_3_ ProRate positively correlates with molecular weight. There was no significant correlation between the selected structural characteristics and the functional indicator, but the indicator at 24 and 48 h showed a high mutual correlation, in agreement with the data shown in Table 2. However, more data are needed to arrive a solid conclusion between these structure–property associations in terms of the modeled parameters.

### 4.8. The Adoption of Framework in Developing a Personalized Nutrition for Infants

Our proposed framework can be utilized as “framework as a service” (FaaS) in commercial settings. Fresh fecal samples from different infants could be collected without harm, followed by In vitro fermentation of a library of selected well-known prebiotics and/or novel prebiotic candidates to evaluate the temporal growth pattern of total probiotic and metabolite production curves. Then, parametric modelling could be applied and growth parameters obtained. Eventually, based on the lag phase, rate, and maximum total probiotic population and/or metabolite yield, tailored prebiotic(s) could be chosen from the candidate library and added to infant formula and/or directly consumed as supplements for breastfed-only infants. Every infant will have their own investigation, and the identified prebiotic(s) will be optimal for that individual infants. We believed this FaaS approach will deliver a value-added application to our proposed framework.

For long-term impact, our framework could be used in healthcare industry when applied to infant patients who need to adopt personalized prebiotic supplements for the alleviation of certain diseases, such as gastrointestinal (GI) discomforts [33,34]. Healthcare practitioners just need to obtain fresh infant fecal samples to obtain the growth and metabolite production parameters in order to select an appropriate prebiotic based on criteria such as a faster growth rate for probiotic colonization to supersede that of pathogens [35], together with slower SCFA production rates to relieve the GI discomfort of infants [36].

### 4.9. Other Possible in-Depth Analysis

Previous publication of parallel investigation of different carbohydrates with time-point measurements of SCFAs (total, acetate, propionate, and butyrate) and dissolved NH_3_, together with the identification of lag phase, maximum increase rate, and maximum production yield in microbial community level, is lacking. Certainly, this is one of the gaps of knowledge. We believed with more data-sharing, a database could be ultimately constructed. Then extra in-depth meta-analysis could be achievable, such as the use of machine learning and deep learning algorithms to predict their interaction, and network simulation to aid novel lead compound identification [37].

### 4.10. Discussion of Data with Literature

Development of prebiotics for infants is different from that of adults as the microbiome between them is very different [38]. Evidence from infant in vivo investigation is promising but it is constrained by safety. On the contrary, In vitro investigation could also be extended to specific human populations such as infants, with the addition of digestion modeling after validation [39]. This approach could also be incorporated into our framework, greatly facilitating the manufacture of tailored or personalized prebiotic supplements.

Dietary fibre can regulate the yield and molar ratios of SCFA metabolites. Acetic and butyric acids are produced mainly by fermentation of aldehydes (e.g., glucose, galactose, mannose, and xylose), whereas propionic acid is produced mainly by ketone (e.g., fructose, arabinose, and tagatose) fermentation and an increase of butyric acid production by xylan, beta-glucan, RS type 3, and RS type 4 [40]. Our data also suggests similar findings with Lactose and GOS producing relatively more acetate in Figure S2a,b. Fructans such as FOS produced the highest level of propionate in Figure S2c and Inulin gave most propionate in Figure S2d. Barley_bG as beta-glucans produced the highest level of butyrate in Figure S2e,f.

In terms of fermentation, beta-glucans from barley has the greatest potential as a novel prebiotic candidate for further development. Compared to classical prebiotics such as oligomeric GOS, polymeric beta-glucans allow more capacity for engineering, such as structural modification to optimize their prebiotic properties.

In addition, parametric modeling gives biological meaning parameters such as the lag maximum rate and maximum capacity of SCFAs and dissolved NH_3_, as well as the highest growth and decline of probiotics. Although there is some literature showing the SCFA production curve against time and discussing the slope of inclination by visual comparison [41], the availability of lag and maximum production rate is scarce in literature, and therefore scientists are encouraged to adopt the present framework to include an extra element of time and parametric modeling.

Moreover, the availability of parameters related to the decline phase is less discussed in literature, especially on the parametric modeling of probiotic growth patterns in a community. The growth parameters obtained could not be compared to the parameters identified from pure bacterial culture investigation due to the complex interactions among different microbes in the community, known as microbe–microbe interaction [42]. Therefore, the investigation of probiotic growth parameters (lag phase, maximum growth rate, and maximum population increase), particularly in the context of community scale, is crucial to prebiotic evaluation in fecal microbiome.

## 5. Conclusions

The present In vitro time-course fermentation study of 13 structurally characterized carbohydrates by infant fecal inoculum, as shown in Figure 1, generated a total of 4446 data points, and a further 1092 data points were generated after parametric modeling. In contrast to the conventional approach of data analysis, a new proposed framework, FaaS, incorporating a parametric modelling and a robust functional indicator calculation (SCFAs/NH_3_), was applied to evaluate the results on the microbial community population pattern (commensal bifidobacteria) and metabolite production patterns against time. The advantage of this new approach was demonstrated in obtaining more biological insights than the conventional ones. In particular, FaaS has facilitated evaluation of potential prebiotics (especially beta-glucans) in personalized nutrition in infants and the model study of the complex network in microbiota research. It is anticipated that more data-sharing using this approach can build up a database for meta-analysis by use of machine learning and deep learning algorithms to facilitate commercial development of novel prebiotics.

## Figures and Tables

**Table 1:** Purity, molecular weights, and solubility assessment of the 13 carbohydrates. Purity was accessed by elemental analysis; pure carbohydrate should consist only C, H and O. The presence of N and S indicated the presence of protein. Molecular weight was determined by size exclusion chromatography (SEC) with calibration by external dextran standards.

Carbohydrate	Class	Elemental Analysis ^1,2^			Molecular Weight (kDa) ^3^			Solubility ^4^
		%N	%C	%S	Count	Peak.1	Peak.2	
**Glucose**	simple sugars	0	39.96 ± 0.06	tr	1	1.34		2
**Lactose**		0	40.04 ± 0.04	0	1	2.67		2
**Sucrose**		0	42.13 ± 0.07	0	1	2.71		2
**Xylitol**	sugar alcohol	0	39.51 ± 0.03	0	1	1.95		2
**FOS**	oligo-saccharide	0	41.70 ± 0.162	0	1	1.96		2
**GOS**		0	40.28 ± 0.00	0	1	1.78		2
**XOS**		0	41.13 ± 0.01	0	1	1.65		2
**Inulin**	poly-saccharide	0	39.75 ± 0.01	0	1	4.17		1
**Glycogen**	alpha-glucan	tr	38.70 ± 0.01	tr	1	31,824.74		2
**Starch**		0	38.42 ± 0.01	0	2	1,088,329.50	18.88	1
**Oat_bG**	beta-glucan	2.12 ± 0.32	38.84 ± 0.27	0.08 ± 0.08	1	1.60		2
**Barley_bG**		0	39.78 ± 0.01	0	1	276.54		2
**Ptr_bG**		0	39.36 ± 0.09	0	2	132.98	2.33	1

^1^ the oxygen element cannot be determined via the instrumental analysis as pure oxygen was supplied for complete oxidation of samples. The hydrogen element is not shown here. ^2^ tr: trace amounts ≤ 0.01%. ^3^ the molecular weight determination of carbohydrates calibrated with external dextran standards, which are a linear chain of alpha-glucans. ^4^ Solubility was determined by visual inspection in culture medium (see following sections) after sterilization and denoted as “2” for soluble, “1” for colloidal, and “0” for insoluble.

**Table 2:** Different test statistics of total short-chain fatty acids, dissolved ammonia, and the SCFAs/NH_3_ ratio at 24 and 48 h. Robustness of indicator could capture most of the essential information at both 24 and 48 h to allow a rough but valid comparison between the two time-points in meta-analysis.

Test Statistics	Indicator		
	Total SCFAs	Dissolved NH_3_	SCFAs/NH_3_ Ratio
**Distribution**			
24-48 h change dispersion ^1^	0.964	0.953	0.743 *
Paired Wilcoxon rank sum ^2^, *p*-value	0.001	0.033	0.376 *
Paired *t*-test, *p*-value	0.004	0.029	0.251 *
**Correlation**			
Spearman correlation, rho	0.610	0.709 *	0.709 *
Spearman correlation, *p*-value	0.030	0.009 *	0.009 *
Pearson correlation, coefficient	0.883	0.805	0.894 *
Pearson correlation, *p*-value	6.222 × 10^−5^	9.093 × 10^−4^	3.780 × 10^−5^ *
**Similarity ^3^**			
Euclidean	0.374	0.316	0.385 *
Manhattan	0.176	0.143	0.178 *
Cosine	0.883	0.805	0.894 *

* denotes the best performer among the three; ties can occur. ^1^ The dispersion is calculated based on the absolute value of slope between time 24 and 48 h to capture the magnitude of change between this 24-h period, i.e., (|slope|.sd/|slope|.mean). ^2^ Paired test statistics were used capture the change of each carbohydrate at 24 and 48 h. ^3^ Similarity has a range from 0 to 1; the data is z-score standardized before calculating the similarity values. Euclidean distance: consider both magnitude and direction; Manhattan distance: consider only the magnitude; cosine distance: consider only the direction.

## Supplementary Materials

The following are available online at https://www.mdpi.com/2076-2607/8/5/623/s1.
