# Supplementary material for: Framework as a Service, FaaS: Personalized Prebiotic Development for Infants with the Elements of Time and Parametric Modelling of In Vitro Fermentation
Source: Microorganisms. 2020 Apr 25;8(5):623. doi: 10.3390/microorganisms8050623 (PMC7285508; doi:10.3390/microorganisms8050623)

Figure S1: Four selected short chain fatty acid profiles of GC-FID chromatograms. **(a)** Short chain fatty acids standards including acetic, propionic, butyric, valeric and caproic acids. IS refer to the "internal standard", which was methyl-valeric acid; **(b)** Inulin at timepoint 205 h with propionic acid as the most dominant SCFA; **(c)** Barley_bG at timepoint 205 h with butyric acid as the most dominant SCFA; **(d)** GOS at timepoint 205 h with acetic acid as the most dominant SCFA. Most SCFAs were eluted with a retention time between 7 to 17 min.

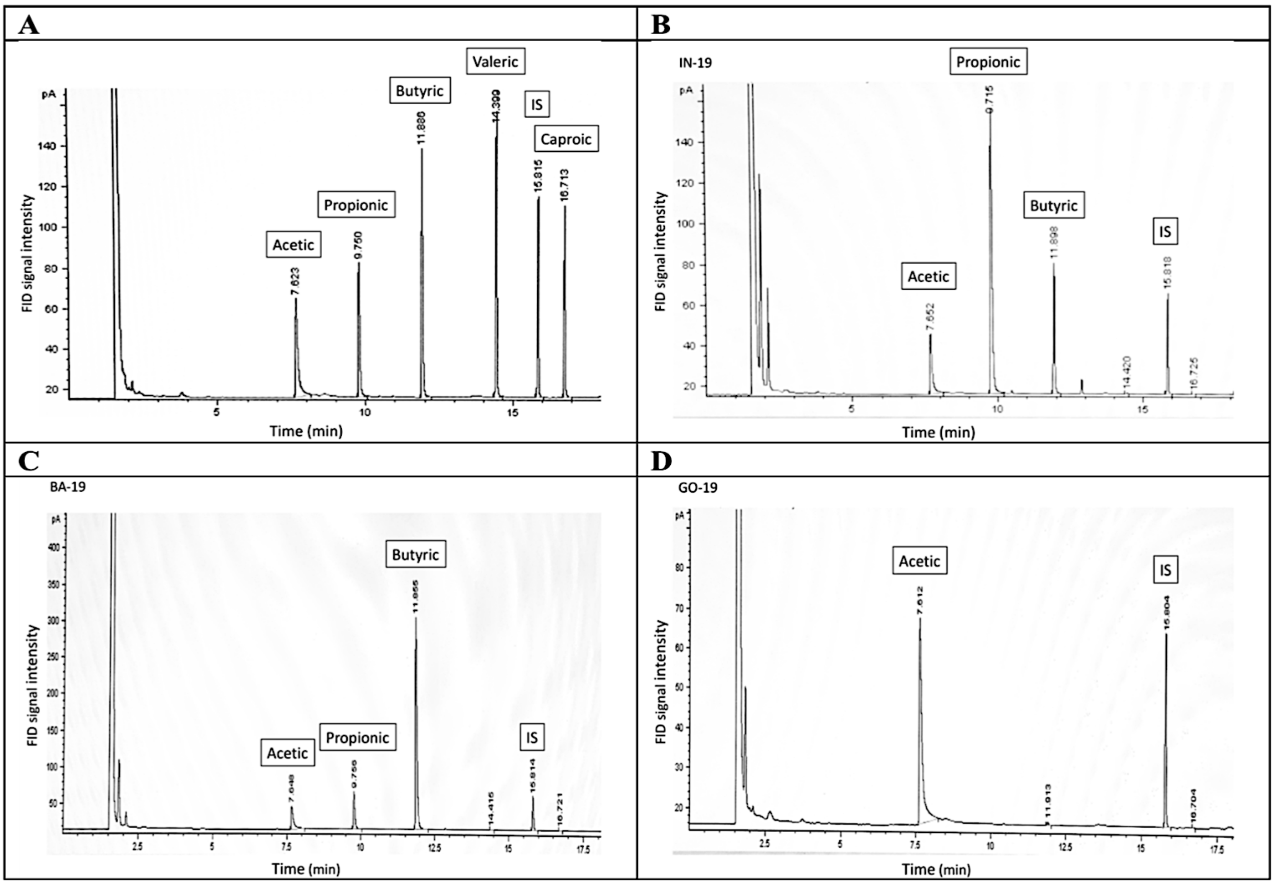

Supplement: Supplementary file 1 [file microorganisms-08-00623-s001.zip › FigureS1.pdf]
