# Supplementary material for: Framework as a Service, FaaS: Personalized Prebiotic Development for Infants with the Elements of Time and Parametric Modelling of In Vitro Fermentation
Source: Microorganisms. 2020 Apr 25;8(5):623. doi: 10.3390/microorganisms8050623 (PMC7285508; doi:10.3390/microorganisms8050623)

Figure S2: Individual short chain fatty acids produced by fermentation of carbohydrates using infant faecal inoculum. **(a)** Acetic acid at time = 24 hour; **(b)** acetic acid at time = 48 hour; **(c)** propionic acid at time = 24 hour; **(d)** propionic acid at time = 48 hour; **(e)** butyric acid at time = 24 hour; **(f)** butyric acid at time = 48 hour.

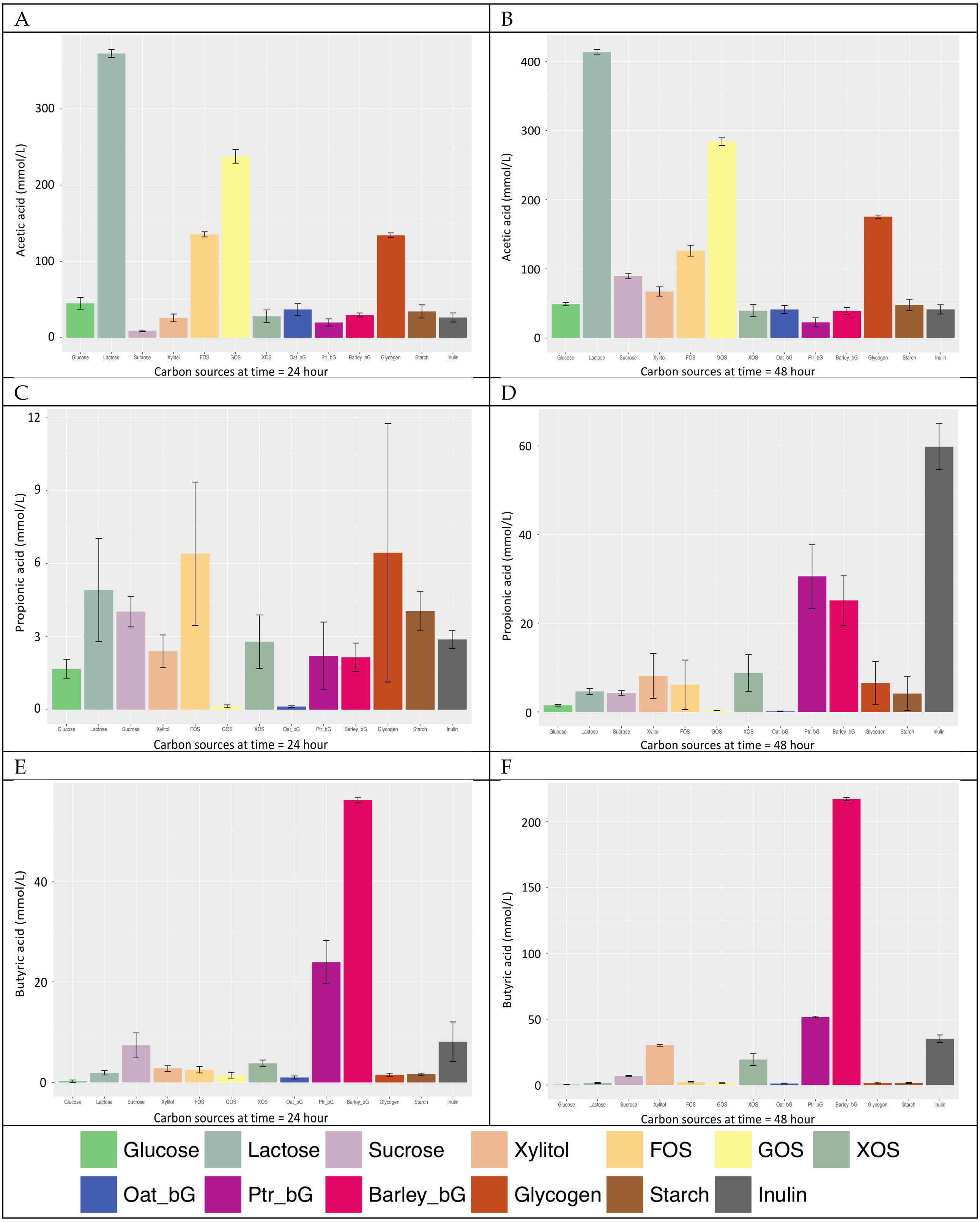

Supplement: Supplementary file 1 [file microorganisms-08-00623-s001.zip › FigureS2.pdf]
