# Supplementary material for: Framework as a Service, FaaS: Personalized Prebiotic Development for Infants with the Elements of Time and Parametric Modelling of In Vitro Fermentation
Source: Microorganisms. 2020 Apr 25;8(5):623. doi: 10.3390/microorganisms8050623 (PMC7285508; doi:10.3390/microorganisms8050623)

Figure S3: The proposed functional indicator based on total short chain fatty acids and dissolved ammonia. **(a)** The overall patterns of the proposed indicator along 205-hour experiment with "loess" best fit line from the 13 prebiotic candidates; **(b)** total short chain fatty acids, SCFAs, at 24- and 48-hour of the 13 prebiotic candidates; **(c)** dissolved ammonia, NH_3, at 24- and 48-hour of the 13 prebiotic candidates; **(d)** functional indicator, SCFAs/NH_3 ratio, at 24- and 48-hour of the 13 prebiotic candidates.

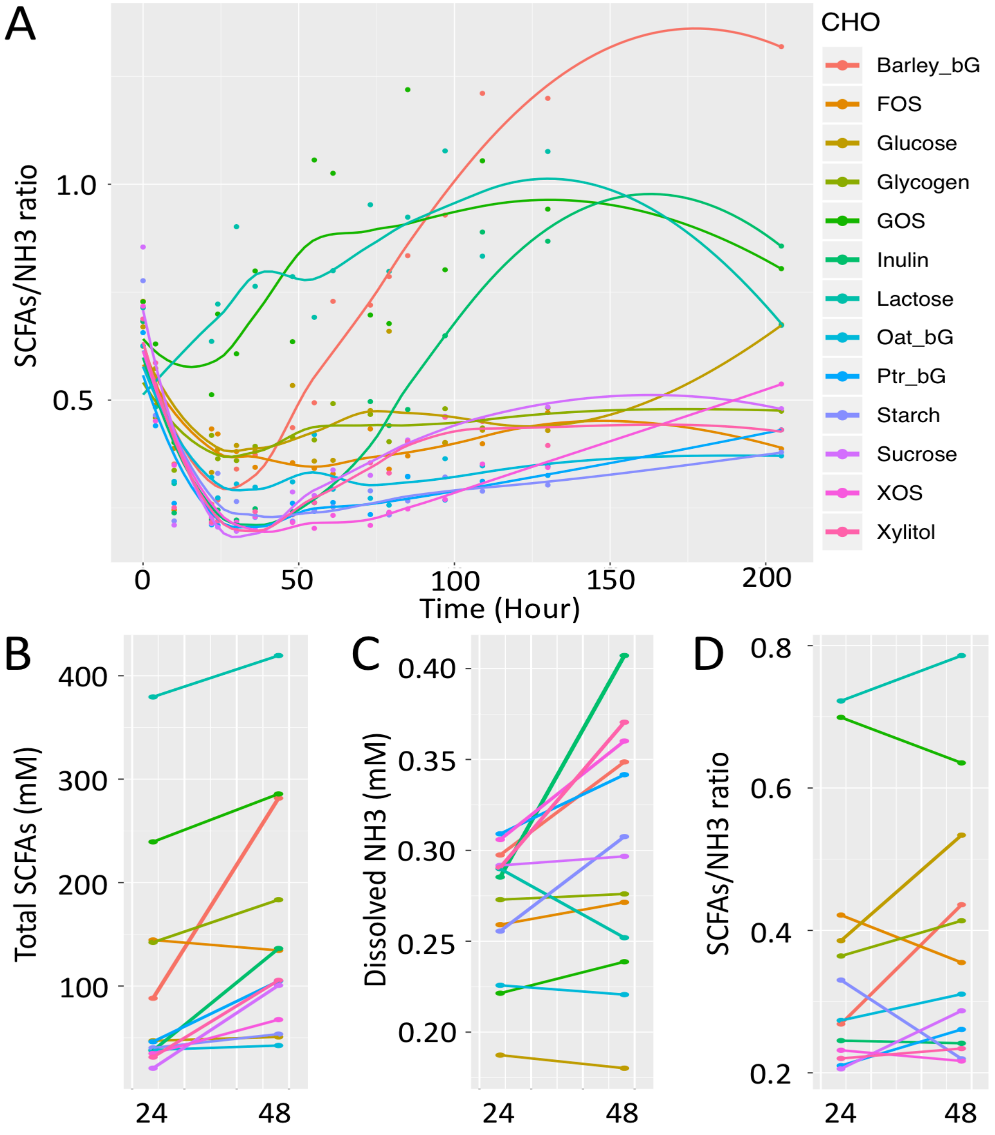

Supplement: Supplementary file 1 [file microorganisms-08-00623-s001.zip › FigureS3.pdf]
