# Supplementary material for: Framework as a Service, FaaS: Personalized Prebiotic Development for Infants with the Elements of Time and Parametric Modelling of In Vitro Fermentation
Source: Microorganisms. 2020 Apr 25;8(5):623. doi: 10.3390/microorganisms8050623 (PMC7285508; doi:10.3390/microorganisms8050623)
Supplement: Supplementary file 1 [file microorganisms-08-00623-s001.zip › TableS1.pdf]

Table S1. Parametric model fitted and the modelled parameters of short chain fatty acids profile. Short chain fatty acids (SCFAs), include acetate, propionate, butyrate and total SCFA of the 13 carbohydrates fermentation temporal patterns. ProRate: maximum production rate; Lag: lag phase in SCFAs production; MaxConc: maximum SCFAs concentration increase. Data are provided as mean and standard deviation for each combination of carbohydrates and SCFAs.

CHO	SCFAs	model	ProRate(mM/hour)	ProRate_sd	Lag(hour)	Lag_sd	MaxConc(mM)	MaxConc_sd
barley_bG	All	gompertz	14.468	0.833	26.932	2.248	1236.164	39.933
FOS	All	gompertz	6.225	1.312	3.765	3.092	156.919	5.463
glucose	All	gompertz	1.054	0.245	-12.075	7.55	65.669	3.929
glycogen	All	gompertz	5.288	0.73	0.609	0.611	219.502	6.548
GOS	All	gompertz	7.537	1.234	1.269	0.836	378.587	15.556
inulin	All	logistic	14.261	1.414	50.711	3.347	959.384	43.494
lactose	All	gompertz	17.534	3.945	5.546	3.718	482.193	17.918
oat	All	gompertz	1.927	0.538	-1.561	0.711	49.162	2.096
ptr	All	gompertz	2.346	0.196	10.424	5.235	410.91	40.472
starch	All	gompertz	1.002	0.117	-9.853	6.544	128.048	10.143
sucrose	All	logistic	4.073	0.314	28.991	2.573	249.832	7.254
XOS	All	logistic	3.678	0.271	55.45	4.501	563.074	47.432
xylitol	All	logistic	5.396	0.498	29.511	2.689	286.651	8.44
barley_bG	Acetic	gompertz	1.275	0.085	12.824	4.137	223.488	17.822
FOS	Acetic	gompertz	6.045	1.302	4.667	3.13	147.383	5.115
glucose	Acetic	gompertz	1.093	0.259	-9.514	7.045	62.574	3.674
glycogen	Acetic	gompertz	5.089	0.704	1.276	0.61	209.995	6.235
GOS	Acetic	gompertz	7.547	1.246	1.486	0.837	375.826	15.458
inulin	Acetic	logistic	2.17	0.269	31.545	5.44	186.751	12.427
lactose	Acetic	gompertz	17.518	3.989	5.892	3.73	474.295	17.556
oat	Acetic	gompertz	2.031	0.596	-0.634	0.677	47.545	2.021
ptr	Acetic	gompertz	0.356	0.053	-21.3	12.042	77.927	15.369
starch	Acetic	gompertz	0.934	0.106	-7.125	6.293	119.196	9.442
sucrose	Acetic	logistic	4.226	0.382	32.411	2.599	221.387	6.457
XOS	Acetic	logistic	0.895	0.085	34.264	12.042	211.675	44.362
xylitol	Acetic	logistic	4.169	0.452	29.631	3.094	216.359	7.292
barley_bG	Butyric	gompertz	10.73	0.653	26.614	2.285	869.024	28.232
FOS	Butyric	logistic	0.059	0.018	-14.667	8.181	2.628	0.115
glucose	Butyric	gompertz.exp	NA	NA	NA	NA	NA	NA
glycogen	Butyric	gompertz	0.025	0.007	-36.019	14.195	1.842	0.089
GOS	Butyric	gompertz	0.014	0.003	-70.879	20.877	2.158	0.164
inulin	Butyric	logistic	3.685	0.796	61.062	6.813	239.739	23.902
lactose	Butyric	gompertz	0.013	0.005	-87.648	40.554	2.68	0.46
oat	Butyric	logistic	0.005	0.001	-178.023	48.985	1.638	0.194
ptr	Butyric	gompertz	2.241	0.661	18.405	3.453	56.913	2.433
starch	Butyric	logistic	0.016	0.003	-55.213	21.387	3.336	0.504
sucrose	Butyric	logistic	0.196	0.043	30.229	13.338	27.703	4.856
XOS	Butyric	gompertz	3.227	2.68	277.227	130.946	3016.806	3303.965
xylitol	Butyric	logistic	1.083	0.222	33.642	5.805	55.934	3.678
barley_bG	Propionic	logistic	3.396	0.356	45.142	2.818	165.474	5.794
FOS	Propionic	logistic	1.486	1.033	5.192	2.979	6.571	0.246
glucose	Propionic	logistic	0.011	0.003	-104.339	32.062	2.299	0.186
glycogen	Propionic	logistic	1.584	0.811	5.075	2.208	7.296	0.217
GOS	Propionic	logistic	0.005	0.001	-20.472	9.641	0.551	0.04
inulin	Propionic	logistic	9.263	0.759	54.316	2.384	528.63	17.238
lactose	Propionic	logistic	0.475	0.225	1.279	0.586	5.37	0.229
oat	Propionic	logistic	0	0	-262.348	125.664	0.231	0.063
ptr	Propionic	gompertz	1.651	0.151	36.441	4.894	272.079	30.212
starch	Propionic	gompertz	0.737	0.372	4.183	2.337	5.077	0.204
sucrose	Propionic	gompertz	0.132	0.043	-2.668	1.303	4.98	0.324
XOS	Propionic	gompertz	2.381	0.276	65.7	4.15	249.709	19.241
xylitol	Propionic	logistic	0.194	0.038	12.722	7.727	14.349	1.13
