# Supplementary material for: Framework as a Service, FaaS: Personalized Prebiotic Development for Infants with the Elements of Time and Parametric Modelling of In Vitro Fermentation
Source: Microorganisms. 2020 Apr 25;8(5):623. doi: 10.3390/microorganisms8050623 (PMC7285508; doi:10.3390/microorganisms8050623)
Supplement: Supplementary file 1 [file microorganisms-08-00623-s001.zip › TableS2.pdf]

Table S2. Parametric model fitted and the modelled parameters of dissolved ammonia, NH_3. ProRate: maximum production rate; Lag: lag phase in NH_3 production; MaxConc: maximum NH_3 concentration increased. Data are provided as mean and standard deviation for each combination of carbohydrates and NH_3.

CHO	model	ProRate(mg/mL/hour)	ProRate_sd	Lag(hour)	Lag_sd	MaxConc(mg/mL)	MaxConc_sd
barley_bG	gompertz	0.008539	0.001142	-9.407	3.44	0.407	0.01
FOS	logistic	0.022895	0.01004	-1.139	0.575	0.289	0.01
glycogen	logistic	0.020539	0.003383	-2.301	1.21	0.289	0.038
glucose	logistic	0.008867	0.001601	-7.473	2.162	0.189	0.012
GOS	logistic	0.009539	0.001119	-6.328	1.476	0.227	0.023
inulin	gompertz	0.009379	0.000952	-9.104	2.516	0.43	0.079
lactose	logistic	0.020598	0.008833	-1.761	0.603	0.279	0.01
oat	gompertz	0.007106	0.003736	-11.577	2.044	0.242	0.015
ptr	gompertz	0.00818	0.001071	-13.089	3.766	0.426	0.101
starch	logistic	0.034657	0.011778	0.33	0.281	0.298	0.066
sucrose	logistic	0.030926	0.006854	0.691	0.222	0.303	0.053
XOS	gompertz	0.008602	0.001222	-11.346	3.624	0.398	0.096
xylitol	gompertz	0.012057	0.001541	-5.794	1.999	0.365	0.065
