# Supplementary material for: Framework as a Service, FaaS: Personalized Prebiotic Development for Infants with the Elements of Time and Parametric Modelling of In Vitro Fermentation
Source: Microorganisms. 2020 Apr 25;8(5):623. doi: 10.3390/microorganisms8050623 (PMC7285508; doi:10.3390/microorganisms8050623)
Supplement: Supplementary file 1 [file microorganisms-08-00623-s001.zip › TableS3.pdf]

Table S3. Additive parametric models fitted and the modelled parameters of total probiotic population. The model is composed of two addition models. GroRate: maximum total probiotic growth rate; Lag: lag phase; MaxCFU: maximum total probiotic population increase; t_x: tipping point; DeclineRate: maximum population decline rate in the decline phase; DeclineLag: lag phase of the decline phase, representing the sustainability; DeclineMaxCFU: maximum total probiotic population decrease from MaxCFU. Data are provided as mean and standard deviation for each carbohydrate.

CHO	Gro.model	GroRate(CFU/hour)	GroRate_sd	Lag(hour)	Lag_sd	MaxCFU(CFU)	MaxCFU_sd	tx(hour)	Decline.model	DeclineRate(CFU/hour)	DeclineRate_sd	DeclineLag(hour)	DeclineLag_sd	DeclineMaxCFU(CFU)	DeclineMaxCFU_sd
barley_bG	gompertz	323577	54915	-3.053	3.755	13397269	430550	49	gompertz	243100	57393	-6.214	6.511	12783905	642282
FOS	logistic	3151759	123668	6.891	0.122	19370531	14776	22	logistic	398844	33788	61.414	2.119	19686610	422486
glycogen	logistic	6168639	129400	5.413	0.076	20620064	14121	22	gompertz	768132	109460	75.135	1.712	20307328	561119
glucose	gompertz	3864303	1626769	4.728	1.961	19419555	418807	49	logistic	509859	109487	47.542	4.949	20631162	1019025
GOS	logistic	6212723	60161	5.694	0.037	18690008	4256	22	gompertz	429131	65358	72.671	2.967	18816852	741785
inulin	logistic	4764834	283188	6.802	0.190	21090019	17881	22	gompertz	158792	28165	-30.317	11.829	22066977	1762883
lactose	logistic	7628096	2734233	5.039	1.864	13930115	11217	22	gompertz	877039	5614	91.859	0.045	13611321	10080
oat_bG	logistic	11482547	4595632	5.069	2.142	20310127	14199	10	logistic	576199	101107	49.089	3.284	18986417	680848
ptr_bG	gompertz	2603498	590055	4.898	1.104	18036055	186696	49	logistic	921602	563748	12.043	7.270	15789795	862328
starch	gompertz	5493720	2515862	4.830	2.137	24398085	455172	49	logistic	485511	70859	24.057	4.300	25769021	941953
sucrose	gompertz	873362	160935	-2.002	3.203	28470397	880793	49	logistic	1072067	189136	54.411	2.882	28951961	969204
XOS	logistic	5129119	1279393	6.388	4.496	21305007	505656	49	gompertz	920967	189349	7.623	3.047	21244251	582666
xylitol	gompertz	621769	98584	-1.847	3.377	24615515	729690	49	gompertz	1313764	318506	47.474	2.236	24635957	775826
