# Supplementary material for: Framework as a Service, FaaS: Personalized Prebiotic Development for Infants with the Elements of Time and Parametric Modelling of In Vitro Fermentation
Source: Microorganisms. 2020 Apr 25;8(5):623. doi: 10.3390/microorganisms8050623 (PMC7285508; doi:10.3390/microorganisms8050623)
Supplement: Supplementary file 1 [file microorganisms-08-00623-s001.zip › TableS4.pdf]

Table S4. Total short chain fatty acids, dissolved ammonia, total probiotic population and robust functional indicator at 24-hour and 48-hour of the 13 carbohydrates. Data are provided as mean and standard deviation for each carbohydrate.

Carbohydrate	Targets	Hour	Mean	sd
Glucose	SCFAs	24	47.073	2.762
Lactose	SCFAs	24	379.501	2.595
Sucrose	SCFAs	24	20.527	1.349
Xylitol	SCFAs	24	31.264	2.138
FOS	SCFAs	24	144.467	2.310
GOS	SCFAs	24	239.387	3.217
XOS	SCFAs	24	34.774	3.353
Oat_bG	SCFAs	24	38.222	2.639
Ptr_bG	SCFAs	24	46.155	3.482
Barley_bG	SCFAs	24	88.174	1.258
Glycogen	SCFAs	24	142.249	2.923
Starch	SCFAs	24	40.233	3.226
Inulin	SCFAs	24	37.566	3.424
Glucose	SCFAs	48	50.821	0.925
Lactose	SCFAs	48	419.498	1.606
Sucrose	SCFAs	48	100.656	1.650
Xylitol	SCFAs	48	105.461	4.180
FOS	SCFAs	48	134.428	4.674
GOS	SCFAs	48	285.751	1.924
XOS	SCFAs	48	67.505	5.780
Oat_bG	SCFAs	48	42.484	2.102
Ptr_bG	SCFAs	48	104.804	4.823
Barley_bG	SCFAs	48	281.636	3.968
Glycogen	SCFAs	48	183.434	2.597
Starch	SCFAs	48	53.504	4.142
Inulin	SCFAs	48	136.274	4.927
Barley_bG	NH3	24	0.297	0.046
FOS	NH3	24	0.259	0.014
Glycogen	NH3	24	0.187	0.012
Glucose	NH3	24	0.273	0.069
GOS	NH3	24	0.221	0.015
Inulin	NH3	24	0.285	0.017
Lactose	NH3	24	0.290	0.017
Oat_bG	NH3	24	0.226	0.029
Ptr_bG	NH3	24	0.309	0.067
Starch	NH3	24	0.256	0.020
Sucrose	NH3	24	0.292	0.015
XOS	NH3	24	0.306	0.019
Xylitol	NH3	24	0.291	0.015
Barley_bG	NH3	48	0.349	0.011
FOS	NH3	48	0.271	0.054
Glycogen	NH3	48	0.180	0.016
Glucose	NH3	48	0.276	0.034
GOS	NH3	48	0.239	0.015
Inulin	NH3	48	0.407	0.010
Lactose	NH3	48	0.252	0.056
Oat_bG	NH3	48	0.221	0.012
Ptr_bG	NH3	48	0.342	0.014
Starch	NH3	48	0.308	0.062
Sucrose	NH3	48	0.297	0.091
XOS	NH3	48	0.360	0.017
Xylitol	NH3	48	0.370	0.084

Barley_bG	Probiotic	24	8320000	19998
FOS	Probiotic	24	18300000	377285
Glycogen	Probiotic	24	19770000	580752
Glucose	Probiotic	24	17020000	124600
GOS	Probiotic	24	10540000	147700
Inulin	Probiotic	24	19660000	122249
Lactose	Probiotic	24	11890000	491024
Oat_bG	Probiotic	24	16260000	143900
Ptr_bG	Probiotic	24	18370000	148964
Starch	Probiotic	24	22980000	170100
Sucrose	Probiotic	24	21770000	726724
XOS	Probiotic	24	16860000	985399
Xylitol	Probiotic	24	16810000	202900
Barley_bG	Probiotic	48	13280000	901807
FOS	Probiotic	48	7530000	11840
Glycogen	Probiotic	48	20030000	1322
Glucose	Probiotic	48	1030000	175
GOS	Probiotic	48	3160000	30006
Inulin	Probiotic	48	14970000	962748
Lactose	Probiotic	48	300	3
Oat_bG	Probiotic	48	18370000	1054
Ptr_bG	Probiotic	48	18350000	149899
Starch	Probiotic	48	25410000	189280
Sucrose	Probiotic	48	28540000	311603
XOS	Probiotic	48	22120000	1096
Xylitol	Probiotic	48	24470000	180061
Barley_bG	SCFAs/NH3	24	0.269	0.036
FOS	SCFAs/NH3	24	0.422	0.126
Glucose	SCFAs/NH3	24	0.386	0.133
Glycogen	SCFAs/NH3	24	0.364	0.022
GOS	SCFAs/NH3	24	0.699	0.291
Inulin	SCFAs/NH3	24	0.245	0.091
Lactose	SCFAs/NH3	24	0.722	0.274
Oat_bG	SCFAs/NH3	24	0.274	0.050
Ptr_bG	SCFAs/NH3	24	0.210	0.019
Starch	SCFAs/NH3	24	0.330	0.211
Sucrose	SCFAs/NH3	24	0.206	0.055
XOS	SCFAs/NH3	24	0.232	0.098
Xylitol	SCFAs/NH3	24	0.220	0.057
Barley_bG	SCFAs/NH3	48	0.436	0.035
FOS	SCFAs/NH3	48	0.355	0.039
Glucose	SCFAs/NH3	48	0.534	0.393
Glycogen	SCFAs/NH3	48	0.414	0.070
GOS	SCFAs/NH3	48	0.635	0.149
Inulin	SCFAs/NH3	48	0.242	0.016
Lactose	SCFAs/NH3	48	0.786	0.063
Oat_bG	SCFAs/NH3	48	0.311	0.087
Ptr_bG	SCFAs/NH3	48	0.261	0.038
Starch	SCFAs/NH3	48	0.220	0.023
Sucrose	SCFAs/NH3	48	0.287	0.017
XOS	SCFAs/NH3	48	0.217	0.053
Xylitol	SCFAs/NH3	48	0.234	0.018
